# Endoport-Assisted Microsurgical Treatment of a Ruptured Periventricular Aneurysm

**DOI:** 10.1155/2016/8654262

**Published:** 2016-04-19

**Authors:** Ching-Jen Chen, James Caruso, Robert M. Starke, Dale Ding, Thomas Buell, R. Webster Crowley, Kenneth C. Liu

**Affiliations:** ^1^Department of Neurological Surgery, University of Virginia Health System, Charlottesville, VA 22908, USA; ^2^Department of Radiology & Medical Imaging, University of Virginia Health System, Charlottesville, VA 22908, USA

## Abstract

*Background and Importance.* Ruptured periventricular aneurysms in patients with moyamoya disease represent challenging pathologies. The most common methods of treatment include endovascular embolization and microsurgical clipping. However, rare cases arise in which the location and anatomy of the aneurysm make these treatment modalities particularly challenging.* Clinical Presentation.* We report a case of a 34-year-old female with moyamoya disease who presented with intraventricular hemorrhage. CT angiography and digital subtraction angiography revealed an aneurysm located in the wall of the atrium of the right lateral ventricle. Distal endovascular access was not possible, and embolization risked the sacrifice of arteries supplying critical brain parenchyma. Using the BrainPath endoport system, the aneurysm was able to be accessed. Since the fusiform architecture of the aneurysm prevented clip placement, the aneurysm was ligated with electrocautery.* Conclusion.* We demonstrate the feasibility of endoport-assisted approach for minimally invasive access and treatment of uncommon, distally located aneurysms.

## 1. Background and Importance

Patients with moyamoya disease are at risk for developing aneurysms. Moyamoya disease is characterized by progressive stenosis of the internal carotid artery with subsequent development of convoluted collateral vessels [1]. Dysregulation of cerebral blood flow is thought to contribute to aneurysm formation in a variety of locations and increased susceptibility to ischemia and hemorrhage. Yamashita et al. theorize that moyamoya patients are predisposed to hemorrhage due to weakened vessel media and increased fibrosis of collateral vessels, which are physiologically primed for aneurysm formation [2]. Aneurysms of collateral vessels are often in deep locations, such as the basal ganglia and periventricular white matter [3].

Conventional microsurgical approaches for the treatment of these deeply located aneurysms can lead to white matter injury and ischemia of surrounding parenchyma. Prolonged retraction can predispose patients to seizures, venous infarction, and swelling [4, 5]. Endovascular embolization offers another treatment modality, although vessel tortuosity may preclude access to these distal aneurysms.

Development of minimally invasive techniques has been proposed to reduce complications associated with brain retraction. In particular, endoport-assisted microsurgery has shown promising results in minimizing complications associated with treatment of deep cerebral pathology, including periventricular lesions [4, 6–8]. We present a novel application of an endoport system for the treatment of a ruptured periventricular aneurysm in a patient with moyamoya disease.

## 2. Clinical Presentation

**Figure 1:**
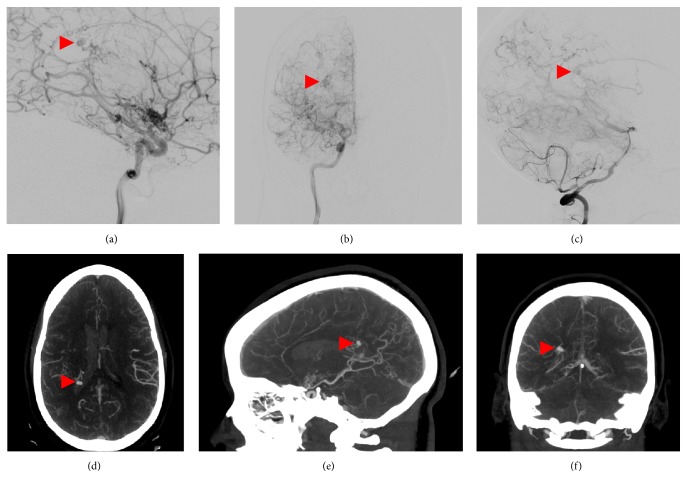
Cerebral angiography, (a) lateral and (b) AP projections of a right internal carotid artery injection and (c) lateral projection of a right vertebral artery injection, shows an aneurysm (arrowhead) arising from the medial posterior choroidal artery branch of the right posterior cerebral artery, with stagnation of contrast in the late arterial phase. Preoperative CTA, (d) axial, (e) sagittal, and (f) coronal views, shows a 5 × 4 × 3 mm periventricular aneurysm (arrowhead), projecting into the atrium of the right lateral ventricle with multiple adjacent collateral vessels.

A 34-year-old female with a moyamoya disease and a prior history of intraventricular hemorrhage (IVH) presented to an outside hospital with headache, nausea, and vomiting. Brain computed tomography (CT) showed significant IVH. Subsequent CT angiogram (CTA) and digital subtraction angiography (DSA) (Figures 1(a)–1(c)) demonstrated a periventricular aneurysm in the wall of the atrium of the right lateral ventricle supplied by tortuous vessels arising from the posterior choroidal artery. The patient was subsequently transferred to our institution for further management and was neurologically nonfocal on arrival. The patient was then taken to the neurointerventional suite after a preoperative CTA (Figures 1(d)–1(f)). However, distal access to the aneurysm could not be safely achieved via endovascular approach, and embolization could not be performed without sacrificing the arterial supply to the adjacent brain parenchyma. Super-selective catheterization of the posterior choroidal artery demonstrated that significant parenchyma was supplied by distal collaterals. Therefore, we elected to proceed with endoport-assisted microsurgical treatment of the aneurysm.

The techniques of endoport-assisted microsurgery at our institution have been described previously [4]. Briefly, the patient was placed into three-point cranial fixation in the prone position using a Mayfield Skull Clamp system (Integra, Plainsboro, NJ, USA), and the StealthStation frameless stereotactic neuronavigation system (Medtronic, Minneapolis, MN, USA) was used to determine the optimal trajectory for direct visualization of the aneurysm from a transcortical parietal approach. A craniotomy over the planned entry point, measuring approximately 4 cm in diameter, was created and a small cruciate dural opening was made.

**Figure 2:**
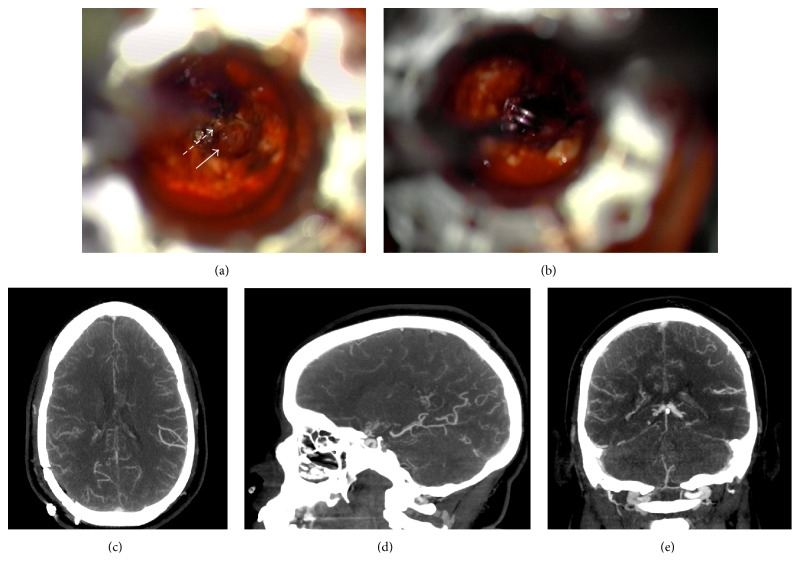
(a) Intraoperative view through the outer sheath of the BrainPath endoport system shows exposure of the proximal vessel (dashed arrow) and the partially thrombosed, fusiform periventricular aneurysm (solid arrow) within the atrium of the right lateral ventricle. (b) A temporary clip was placed on the inflow vessel to achieve proximal control prior to ligation of the aneurysm with electrocautery. Postoperative CTA, (c) axial, (d) sagittal, and (e) coronal views, shows interval decrease in the quantity of intraventricular hemorrhage and no evidence of residual aneurysm.

The BrainPath endoport system (NICO Corporation, Indianapolis, IN, USA) was used for the approach. The endoport system consists of an outer sheath and an inner obturator. The outer sheath is 13.5 mm in diameter with variable lengths of 50 mm, 60 mm, and 75 mm. The sheath is left in place once desired position is obtained and allows for fixation to a Greenberg retractor system. The inner obturator has a blunt, tapered tip which extends 15 mm beyond the outer sheath and allows for gradual cannulation of the brain parenchyma by the endoport system. Under constant image guidance, a 50 mm BrainPath sheath was advanced along the planned route into the atrium of the right lateral ventricle. Acute hematoma under moderately high pressure was encountered when the inner obturator was removed. This was easily evacuated, and the sheath was advanced further into the atrium. A portion of the ependymal roof of the right lateral ventricle was resected to expose the complex of vessels and the aneurysm. Intraoperatively, the aneurysm appeared larger than expected, likely due to partial thrombosis (Figure 2(a)). However, due to the fusiform architecture of the aneurysm, clip reconstruction could not be successfully performed without occluding the distal outflow. A temporary clip was placed on the inflow vessel to achieve proximal control (Figure 2(d)), and the aneurysm was ligated with electrocautery. The patient was monitored in the intensive care unit postoperatively and was transferred to the regular ward on postoperative day one. Postoperative CT angiography demonstrated reduced intraventricular hemorrhage and no evidence of residual aneurysm (Figures 2(c)–2(e)). The patient was discharged home on postoperative day three.

## 3. Discussion

Periventricular aneurysms are extremely rare, with less than 60 cases reported in the literature, 19 of which were associated with moyamoya disease [9]. Most periventricular aneurysms are believed to develop secondary to malignant hypertension, infection, or arteriovenous malformation, although some are thought to be idiopathic [9]. Despite their deep location, periventricular aneurysms have been treated using modalities similar to those utilized for aneurysms arising from more common locations.

Conservative management has been proposed, since smaller aneurysms may regress spontaneously [9–11], but some cases treated conservatively have resulted in death [9, 12]. Endovascular embolization is a viable option for periventricular aneurysms in moyamoya disease patients as it prevents disruption of collateral vessels and avoids the risk of ischemia and parenchymal damage associated with a microsurgical approach [1]. Patients with deep-seated aneurysms generally respond well to endovascular intervention, although the size of the parent artery is a crucial determinant of success [9]. In a case series investigating patients with moyamoya disease, endovascular embolization resulted in complete obliteration of aneurysms in seven out of eight patients (88%) [3]. Additionally, no complications were observed in the postoperative period, and six patients had full recovery at discharge (75%) [3]. However, successful endovascular embolization in moyamoya patients can be challenging, since the collateral vessels may be tortuous and prone to rupture [1].

Microsurgical clipping has been the traditional treatment for aneurysms, but the risk of intraoperative rupture may be higher when treating deep-seated aneurysms. In addition, craniotomy and prolonged brain retraction can be associated with increased morbidity. Hence, minimally invasive approaches, such as miniature craniotomies and endoscopy, have been utilized for the treatment of deep intracranial pathology, with varying degrees of success.

Compared to other minimally invasive methods available for periventricular aneurysm treatment, endoport-assisted microsurgery is a novel method to gain access to deep intracranial pathology. We present the first report of endoport-assisted treatment of an intracranial aneurysm. The endoport may be associated with a lower risk of retraction-induced complications since the endoport distributes force evenly over surrounding tissues, in contrast to the uneven force applied by the retractor blades in conventional microsurgery [13]. In our case, the endoport also allowed evacuation of the intraventricular blood clot, which may reduce the need for permanent cerebrospinal fluid diversion. Despite these early reports of success with the endoport, the endoport system is limited by its dependence on neuronavigation and an impaired range of motion due to the device's rigidity [4, 5]. Additionally, due to restricted peripheral visualization, endoport use is best applied to pathologies with diameters smaller than that of the endoport [4, 5]. Calcified lesions and those with a diameter greater than 3 cm may significantly hamper resection through an endoport [5]. Despite these caveats, the endoport provides a suitable minimally invasive alternative to conventional microsurgical approaches for the treatment of aneurysms in deep intracranial locations not amenable to endovascular embolization.

## 4. Conclusion

Periventricular aneurysms are rare cerebrovascular entities, although patients with moyamoya disease may be predisposed to developing these lesions. Endoport-assisted microsurgery is a minimally invasive, safe, and effective treatment approach for periventricular and similarly deep-seated intracranial aneurysms.
